# Statistical traces of long-term memories stored in strengths and patterns of synaptic connections

**DOI:** 10.1186/1471-2202-12-S1-O2

**Published:** 2011-07-18

**Authors:** Armen Stepanyants, Gina Escobar

**Affiliations:** 1 Department of Physics, Northeastern University, Boston, MA 02115, USA

## 

Learning and long-term memory rely on plasticity of neural circuits. In adult cerebral cortex plasticity can result from potentiation and depression of synaptic strengths and structural reorganization of circuits through growth and retraction of dendritic spines. By analyzing 166 distributions of spine head volumes and spine lengths from mouse, rat, monkey, and human brains, we determine the “generalized cost” of dendritic spines. This cost universally depends on spine shape, i.e. the dependence is the same in all the analyzed systems. We show that in adult synaptic strength and structural synaptic plasticity mechanisms are in statistical equilibrium, the numbers of dendritic spines in different cortical areas are nearly optimally chosen for memory storage, and the distributions of spine lengths and head volumes are governed by a single parameter – the effective temperature. We suggest that the effective temperature may be viewed as a measure of circuit stability or longevity of stored memories.

